# Electronic Nose and Gas Chromatograph Devices for the Evaluation of the Sensory Quality of Green Coffee Beans

**DOI:** 10.3390/foods13010087

**Published:** 2023-12-26

**Authors:** Gema Cascos, Jesús Lozano, Ismael Montero-Fernández, Jhunior Abrahan Marcía-Fuentes, Ricardo S. Aleman, Antonio Ruiz-Canales, Daniel Martín-Vertedor

**Affiliations:** 1 Technological Institute of Food and Agriculture CICYTEX-INTAEX, Junta of Extremadura, Avda Adolfo Suárez s/n, 06007 Badajoz, Spain; gcascosc01@educarex.es; 2 Industrial Engineering School, University of Extremadura, 06006 Badajoz, Spain; jesuslozano@unex.es; 3 Department of Chemical Engineering and Physical Chemistry, University of Extremadura, Avda de Elvas s/n, 06006 Badajoz, Spain; ismonterof@unex.es; 4 Faculty of Technological Sciences, Universidad Nacional de Agricultura, Catacamas 16201, Honduras; jmarcia@unag.edu.hn; 5 School of Nutrition and Food Sciences, Louisiana State University Agricultural Center, Baton Rouge, LA 70803, USA; rsantosaleman@lsu.edu; 6 Engineering Department, Politechnic High School of Orihuela, Miguel Hernández University of Elche, 03312 Elche, Spain; acanales@umh.es

**Keywords:** green coffee, coffee quality, tasting panel, coffee aroma quality, electronic nose

## Abstract

The aim of this work is to discriminate between the volatile org9anic compound (VOC) characteristics of different qualities of green coffee beans (*Coffea arabica*) using two analysis approaches to classify the fresh product. High-quality coffee presented the highest values for positive attributes, the highest of which being fruity, herbal, and sweet. Low-quality samples showed negative attributes related to roasted, smoky, and abnormal fermentation. Alcohols and aromatic compounds were most abundant in the high-quality samples, while carboxylic acids, pyrazines, and pyridines were most abundant in the samples of low quality. The VOCs with positive attributes were phenylethyl alcohol, nonanal and 2-methyl-propanoic acid, and octyl ester, while those with negative attributes were pyridine, octanoic acid, and dimethyl sulfide. The aroma quality of fresh coffee beans was also discriminated using E-nose instruments. The PLS-DA model obtained from the E-nose data was able to classify the different qualities of green coffee beans and explained 96.9% of the total variance. A PLS chemometric approach was evaluated for quantifying the fruity aroma of the green coffee beans, obtaining an $R_{P}^{2}$ of 0.88. Thus, it can be concluded that the E-nose represents an accurate, inexpensive, and non-destructive device for discriminating between different coffee qualities during processing.

## 1. Introduction

Coffee is one of the most consumed beverages in the world due to its organoleptic characteristics and stimulating effects [1,2].

The botanical family of coffee consists of around 500 genera and more than 6000 species. The most important genus from an economic viewpoint is Coffea, with more than 25 species. About 98% of the nine million tons of green coffee harvested annually are of the Arabica and Robusta varieties.

The two main coffee species have important sensory differences. Arabica is more aromatic, with a moderate body, average bitterness, high acidity, and less caffeine, while Robusta coffee is less aromatic, fuller-bodied, and more bitter [3]. The physical and/or organoleptic qualities of coffee are impacted by climate conditions, soil type, crop management, post-harvest treatment, and grain damage due to insect attacks, such as those of the coffee berry borer [4].

Its originality, strength, and character have inspired much research over the years in search of the principle chemical responsible for its aroma [5]. The particular flavor and aroma of coffee are the result of around a thousand chemical compounds already present in the product [6]. We have to take into account that there are many more volatile compounds that are generated during the roasting process that differ from those of green coffee, such as amino acids and proteins, among others [7]. Furthermore, the chemical composition of the grain changes with the temperature. However, only a small number of these factors contribute to the perceived aroma. The volatile compounds allow us to determine the variety of coffee, the processing techniques used by farmers, its geographical origin and, of course, its quality [8].

The most characteristic volatile compounds belong to the family of furans, pyrroles, pyrazines, ketones, aldehydes, alcohols, acids, pyridines, thiazoles, esters, hydrocarbons, and/or aromatics in both green and roasted coffee [9]. These compounds are determined using laboratory techniques, such as gas chromatography. This technique is widely used for the separation of volatiles, semi-volatiles, and thermally stable components at temperatures of up to 350–400 °C. Gas chromatography establishes the number of individual components present in a sample by using calibration curves of the corresponding standards. Some of these techniques are present in certain laboratories, while other techniques are selected when detecting compounds in different matrixes [10].

A sensory evaluation is another method of analysis that aims to collect, measure, and analyze food properties that are captured by the sense organs [11]. This analysis requires specialized personnel trained in the product to be tasted. A sensory evaluation involves the preparation and tasting of foods and beverages under controlled conditions in such a way that factors that could cause bias are minimized. Attributes such as aroma, flavor, acidity, and body are the sensory characteristics that describe coffee quality [8].

Electronic devices such as electronic noses (E-nose) can be used in the coffee industry to classify green beans of different qualities prior to processing. They are a combination of technology that detects and analyzes chemical compounds in the air and enables the discrimination between agroalimentary food with different aroma profiles, such as wine, citrus [12], fungi [13], or olives, including virgin olive oil [14] and table olives [15,16,17], with the use of matrices. The main technology involved in this work is the E-nose sensors, which are designed to react to specific molecules and generate electrical signals proportional to the detected aroma. When these sensors come into contact with coffee beans, they can discriminate between the type of production based on its quality. The electrical signals generated by the sensors are converted into digital data that can be processed by the system. An important part of E-nose technology is the use of algorithms and machine learning techniques to analyze and process the data obtained from the sensors. These algorithms are essential for the identification of the different odors and chemical compounds present in the coffee bean samples. Finally, the data collected are compared against previously established databases that contain information on the different odors and chemical compounds to enable the identification of the odors and the issuance of results.

An E-nose has a fast response time and is a low-cost device that can be used as an alternative to gas chromatography and sensory evaluation that enables the classification of food according to its odor profile. In an attempt to improve quality and profitability, the coffee industry seeks to implement such engineering advances in all processes, a large part of which has already been documented with regard to establishing quality standards associated with each stage. In this sense, the electronic device could detect the odor characteristics of green coffee, classifying them into different qualities. Furthermore, this equipment could help industries standardize the harvested green coffee before it enters the industry or the roasting process [18], thereby helping control the quality [19,20]. Thus, two different E-noses were used to increase the discriminatory power of the studied fresh coffee samples of different qualities. Therefore, the aim of this work is to demonstrate that this technology is capable of obtaining a data matrix that allows us to establish a correlation between the acquired signals of the electronic sensors of the sensory analysis and volatile organic compounds’ characteristics from coffee samples of different qualities, making it a useful measuring instrument for a first classification.

## 2. Materials and Methods

### 2.1. Samples

Samples of *Coffea arabica* (arabica coffee) were harvested at the red stage of ripeness by a company in the Choluteca region (Honduras). Once collected, they were transported to the plant to be washed and dried using natural processes. Many of the volatile compounds in coffee are generated during the roasting process. This is very different and less numerous in fresh coffee [7]. In addition, it prevents the sensors from being affected by humidity. Green coffee beans were vacuum-packed in portions and stored at room temperature until the analyses were carried out.

### 2.2. Sensory Analysis

The sensory characteristics of the samples were evaluated by a tasting panel made up of eight experts of the CICYTEX research center who were trained in the descriptive analysis of products derived from coffee provided by industry. The tasters had given their informed consent to carry out the olfactory evaluation of the samples studied. This evaluation was performed in different cabins in a tasting room [16]. Green coffee beans (10 g) from different categories were placed into a standard glass jar, covering the bottom of the glass, arranged in a single layer, and kept at 28 °C. Samples were sensory evaluated by the panel to be classified into three different sensory categories: high, medium, and low quality. The positive attributes evaluated by the tasting panel were fruity, herbal, sweet, nutty, and spiced, while the negative ones were roasted, smoky, fermented, and earthy.

The high-quality green coffee beans presented a positive aroma higher than 6 points and a defect attribute less than 1. The medium-quality beans presented a positive aroma between 4 and 6 points and a defect between 2 and 3. The low-quality beans presented a positive aroma of less than 4 and a defect higher than 3 points.

First, a triangular evaluation test was carried out on green coffee beans of different qualities. Two sessions per day and four in one week were conducted. Tasters had to identify which of the three tasting glasses was different. This test allowed us to determine if two samples were significantly different. The minimum number of correct answers was indicated to establish significant differences between the two samples (*p* < 0.05).

Secondly, a descriptive analysis was carried out. The different intensity of positive and negative attributes perceived by the tasters was evaluated on a structured scale from 0–10. The result of each attribute was expressed as average values when the coefficient of variation was less than 20. A total of 33 samples were studied in triplicates. 

### 2.3. Analysis of Volatile Compounds

The profiles of volatile compounds of green coffee beans were analyzed using the method described by Sánchez et al. [15] using static headspace and gas chromatography–mass spectrometry. A sample weighing 2 g was placed into a vial, to which 7 mL of NaCl solution (30% *w*/*v*) was added. A polydimethylsiloxane/divinylbenzene (PDMS/DVB) StableFlex fiber (65 μm, Supelco) was used to sample the volatile compounds. The vials were closed and kept at 40 °C for 30 min according to the methodology described by Sánchez et al. [15]. 

Determinations were performed using gas chromatograph with a triple quadrupole mass spectrometry detector model 456-GC using a capillary column Agilent DB WAXetr (60 m × 0.25 mm; DI: 0.25 mm). Peaks were tentatively identified with the help of the NIST 2.0 MS reference spectral library.

### 2.4. E-Nose Measurements

An E-nose is a piece of miniaturized electronic equipment composed of 11 sensors [15]. It has low power consumption and is the size of a hockey puck, thereby making it easily portable and convenient. The device contains novel metal oxide semiconductor (MOX) chip gas sensors which exhibit global selectivity to odor patterns for each sample: (i) BME680 (Bosch Sensortech GmbH, Reutlingen, Germany): temperature (°C), pressure (hPa), humidity (%RH), and gas measurement (Ω); (ii) SGP30 (Sensirion AG, Stäfa, Switzerland): eCO_2_ (ppm), TVOC (ppb), H_2_(2), and ethanol; (iii) CCS811 (ScioSense B.V., Eindhoven, The Netherlands): eCO_2_ (ppm), TVOC (ppb), and sensor resistance (Ω); and (iv) iAQ-Core (ScioSense B.V., Eindhoven, The Netherlands): eCO_2_ (ppm), TVOC (ppb), and sensor resistance (Ω). 

The electronic analysis required 5 g of green coffee beans to be placed into standard tasting glasses, covered with a watch glass, and placed on a block kept at 25 °C. Eleven measurements were taken per sample. The E-nose was placed on top of the cup and the adsorption phase was carried out to adsorb the headspace of the sample for 60 s. Once this time had elapsed, the E-nose was moved to another empty cup to carry out the desorption phase for 30 s to obtain the baseline. The E-nose received data in one second intervals and eight measurements were carried out for each roasted bean sample. After the analyses were conducted, the microprocessor sent the values obtained to a specific application in an intelligent device (mobile phone) via Bluetooth, and a multivariate analysis was completed with a computer. The algorithm used to characterize the sensor response curves to obtain the raw data for the E-nose was maximum signal value minus minimum 1, plus 100, minus 1. As a result, a vector of data with 11 rows (sensors) for each sample was obtained and a multivariate analysis was performed with the data obtained. The E-nose also had temperature and humidity sensors that enabled the monitorization and correction of the data with compensation algorithms. The results of both analyses were analyzed together to obtain more comprehensive information.

### 2.5. Multivariate Data Analysis

Principal component analysis (PCA), partial least squares discriminant analysis (PLS-DA), and partial least squares (PLS) methods were carried out with the data obtained by the E-nose. The chemometric method was that described by Sánchez et al. [17]. The program used for the multivariate analysis was MATLAB version R2016b, version 9.1 (The Mathworks Inc. Natick, MA, USA) with PLS_Toolbox 8.2.1 (Eigenvector Research Inc., Wenatchee, WA, USA).

### 2.6. Statistical Analysis

An analysis of variance (ANOVA) was carried out to establish statistical differences between the different treatments studied. Next, the Tukey method (univariate analysis) was performed to ascertain the differences between each of the classes studied. SPSS 18.0 software was used for the statistical analysis (SPSS Inc., Chicago, IL, USA).

## 3. Results and Discussion

### 3.1. Sensory Analysis of Green Coffee Beans

First, a sensory analysis was conducted with a triangular test to verify the capacity of the panelist to differentiate samples of different qualities (Table 1). The results showed that tasters were able to discriminate between green coffee beans of different qualities. As can be seen in Table 1, the tasters had to identify which of the three tasting glasses were different. The table shows that the different qualities of the coffee samples were tentatively identified in the different assays carried out. Thus, the tasters were able to classify samples according to their smell. It should be noted that in the triangle test, for samples of maximum quality, the percentage of success was 100%, while for the medium and low quality, this percentage decreased to 95–98%. This is a very interesting result since tasters can discriminate samples based on their olfactory quality.

Subsequently, the coffee samples were selected individually to perform a descriptive evaluation of certain positive and negative attributes of the beans. It is well known that arabica coffee has a higher sensory characteristic that gives it the greatest commercial value. For this reason, green coffee beans were sensorially evaluated descriptively by the tasting panel (Table 2). The panel aimed to define whether the green coffee beans had pleasant or unpleasant attributes that could discriminate samples based on their sensory quality.

The tasting panel can be seen to be very accurate. The main positive attributes were related to fruity, herbal, sweet, nutty, and spiced, while the negative attributes were roasted, smoky, fermented, and earthy. The high-quality coffee presented the highest values of the positive attributes with the highest scores in the fruity attribute, followed by the herbal one. The sweet smell was also an important attribute in these coffee samples with differences between the different qualities. Nutty and spiced odors were only significantly different in the highest-quality samples. 

Regarding the negative attributes, roasted was the most significant, followed by smoky. In this case, the low-quality samples showed higher values of this type of attribute. We have to take into account that the roasted odor is a descriptor related to burnt food, while smoky describes a smell associated with smoke from burning wood [3]. Negative attributes were also found in the low-quality samples related to abnormal fermentation products and those with an earthy or musty odor. The coffee industry elaboration process has possibly brought about the development of certain microorganisms that have spoiled these coffee beans. These results concur with those obtained by Illy and Viani [21] who affirmed that the contact of the fruit with soil favors the development of undesirable microbial fermentation.

The results show that the sensory analysis carried out was able to identify the characteristics of the samples tested. Researchers [9] have evaluated sensory attributes in green coffee from different varieties submitted to different industrial conditions. However, the results obtained in this research can be used as a development strategy for classifying this product in the industry. Therefore, these results highlight the market potential for high-quality green coffee. Although there is wide variation of the coffee quality harvested in Colombia, it is evident that the aroma characteristics of high-quality coffee are far more acceptable than the others. Other researchers [9] have indicated that stinker alteration is produced by microbial fermentation of samples during the elaboration process that produces green coffee seeds with fermented, earthy, or putrid aromas, which makes the quality look diminished. The study findings demonstrate that the sensory analysis successfully identified the specific characteristics of the tested samples. Previous research [9] also evaluated sensory attributes in green coffee from different varieties and under various industrial conditions. These results can serve as a valuable development strategy for product classification in the industry, highlighting the potential for high-quality coffee in the market. 

### 3.2. VOCs of Green Coffee Beans

**Figure 1:**
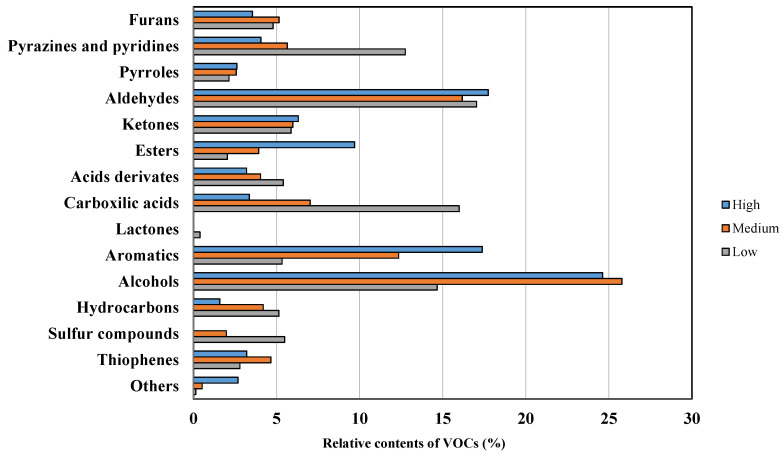
Chemical distribution of volatile compounds (%) in green coffee beans of different qualities.

As can be seen in Figure 1, significant differences were observed between the VOCs of the different green coffee quality analyzed by the GC-MS technique. The families of VOCs that were found in greater proportion were alcohols, aromatics, and aldehydes, while the minor groups were pyrroles, acids derivate, and sulfur compounds.

There are volatiles whose presence gains as the quality of the coffee increases, which could be attributed to the fact that these groups are linked to a positive aroma in coffee beans [9]. That is the case with the alcohol groups, which represented the most abundant compounds in the samples studied. The content of alcohols was greater in the high- and medium-quality beans than in the low-quality beans, which was linked to floral aromas [22]. The aromatic compounds were the second most abundant VOCs in the high-quality samples, with 3.2 times more content compared with the rest of the green coffee beans [23]. Ketones clearly increased to 79.6% and 48.2% in high- and medium-quality coffees, respectively. Similarly, esters increased their content 2.1 times more in the high-quality coffee, although not in the medium-quality group, whose values were slightly lower than in that of the low-quality group. The other group of compounds represented a low percentage in all the qualities and was only moderately higher in the high-quality groups. Moreira et al. [24] obtained similar results.

On the contrary, there were other VOC families that appeared in greater proportion in the low-quality samples. This was the case with carboxylic acids; although they are present in all the samples, their proportion was much higher in the low-quality ones. Their content represented 4.5 times more in the high-quality samples than in the other qualities. This family represented the second most abundant VOCs in low-quality coffees, after the aldehydes group. Pyrazines and pyridines were the third most abundant group in the low-quality coffee. Their content rose 2.0 times more with regard to the high-quality coffee, which had similar values to the medium-quality coffees. These compounds emit negative earthy notes [9,25]. The group of hydrocarbons was the fourth most abundant in the low-quality coffee, representing 7% of the VOCs. They are usually associated with the formation of tar and soot in a cooking or roasting process. Lactones only appeared in low-quality samples in very low proportion. Despite their low concentrations, they were significant in determining the quality of coffee [26]. The odor of this family is associated with a defective odor in seeds [9]. Furthermore, the sulfur compound only appeared in the medium- and low-quality samples, with the greater content in the latter (4.5%). Sulfur compounds are extremely important due to their low odor thresholds. Finally, furans, pyrroles and aldehydes appeared in all the samples and presented no significant differences between the different qualities analyzed. These families appeared in the volatile compound’s literature with roasted or smoked characteristics in different coffee varieties [9,27]. Thiophenes appeared in all the samples but slightly more in those of medium and low quality. These compounds emit negative smoky and roasted notes [9].

The VOCs for the green coffee beans of different quality are shown in Table 3. In total, 70 VOCs were tentatively identified, which were grouped according to different families of VOCs. The main VOCs were phenylethyl alcohol and 2-methoxy-4-vinylphenol, and the minor ones were (E)-7-methyl-1,6-dioxaspiro [4.5], decane, acetic acid, and 2-methyl-butanal.

The furan compounds significantly decreased in low-quality coffee, with 2-methyl-furan and furan representing the most abundant compounds, increasing 2.5 times more than in high-quality coffee. In the literature, these compounds provide negative aromas (smoky or roasted) [28] and are linked to causing mutagenesis in mice [29]; therefore, the European Commission published a recommendation regarding monitoring the presence of this compound in foods [30]. Thus, in green coffee, this family is shown to decrease coffee quality. Pyridine (5.73%) and 2,5-di methyl-pyridine (4.01%) were found in much higher proportion in low-quality coffee in pyrazines and pyrimidines families, with significant results. These VOCs have roasted and burnt notes [21]. As for the group of pyrroles, the main volatile composition varied in the different samples. In medium- and high-quality coffees, 1H-pyrrole (1.12–1.13%) was the main VOC found, and it smells like dried fruit [31]. However, in low-quality coffee, 3,4-dihydro-2,2,3-trimethyl-2H-pyrrole 1-oxide (1.05%) and 1H-indole (0.26%) appeared in low proportion. These compounds are linked to a roasted odor [9]. High contents of pyrazines, pyridines, and pyrroles indicate a decrease in the quality of green coffee. In the group of aldehydes, butanal was the only VOC detected in high-quality coffee (1.28%), while nonanal and 4-hydroxy-3-methoxybenzaldehyde appeared in higher proportions in the medium- and high-quality samples. There were also several compounds that are associated with low-quality coffee, such as 2-octenal, (E)-2-nonenal, and (E,E)-2,4-nonadienal, that appeared exclusively in these samples, and they are associated with buttery fragrances. Hexanal (3.52%) and benzaldehyde (0.65%) were aldehydes found in a higher proportion, and these are markers of poor quality, a rancid odor, and/or oxidative processes [5,9]. The VOCs of the ketone family were found exclusively in the high-quality coffee; specifically, propenone, 1-(4-nitrophenyl)-3-phenylamino (2.73%), and 3-methyl-2-cyclopenten-1-one (1.02%). Both VOCs are associated with sweet smells [4,9]. This family is usually found in coffee of the highest quality. The esters family is linked to positive fruity aromas, mainly due to 3-methylheptyl acetate, propanoic acid, 2-methyl- and octyl ester that appeared in high-quality samples but not in medium- and low-quality ones [4]. The acid derivatives such as 2-methyl-butanoic acid and 3-methyl-2-butenoic acid emit unpleasant odors [32] and only appeared in a greater proportion in the low-quality samples. Regarding the carboxylic acids, nonanoic acid and octanoic acid increased significantly in the low-quality samples. The literature links this family to pungent and rancid odors [32]. γ-Butyrolactone is the only compound within the lactone family that was detected in green coffee beans. Furthermore, it only appeared in the low-quality samples. The literature associates lactones with coffee quality [26] because its odor is linked to a defective odor in seeds [9]. 2-Methoxy-4-vinylphenol and benzene are the main VOCs within the aromatics family, which have a spicy clove odor. The main VOC in the alcohols groups was phenylethyl alcohol, which was the most abundant in green coffee and has a floral smell [33]. Tetradecane, 1-nonene and dodecane, which smell of solvent [4], were the VOCs that appeared in the majority of the hydrocarbons group. These negative attributes only appeared in the low-quality group. Dimethyl sulfide was the only VOC detected in the sulfur compounds family in the low-quality samples. This compound has a putrid or decomposing odor [34]. Finally, in other groups, we must highlight that styrene and beta-pinene presented aromas linked to fresh fruit, citrus, herbs, and green onions [17].

### 3.3. E-Nose Application to Discriminate Quality from Green Coffee Beans

**Figure 2:**
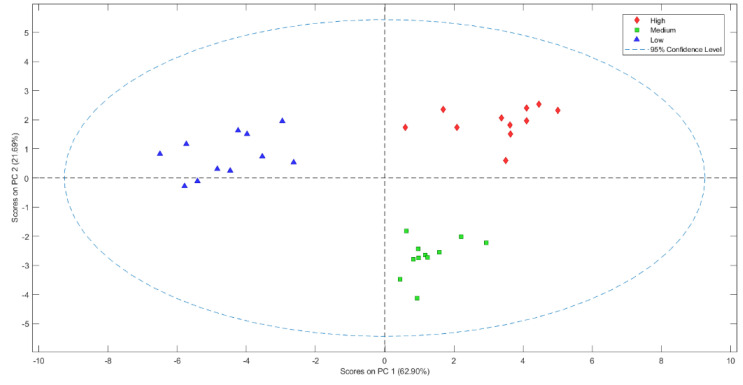
Score plot of the PCA analysis in fresh coffee beans of different qualities.

The volatile compounds of green coffee beans were also evaluated with E-nose devices to discriminate samples based on their quality. The results showed that the PCA was able to identify green coffee beans with different quality categories (Figure 2). The 62.9% of the total variance of the data was explained by PC1, while PC2 explained 21.7%. This is an interesting result since the combined sensors of the electronic devices used were able to discriminate Arabica samples based on their sensory quality. This result could prove useful for companies given that these devices enable a quick and efficient preliminary classification of the quality of the coffee beans before they are subjected to a roasting process. This would allow a quality classification of green coffee to be conducted at the first phases of coffee processing.

Next, a PLS-DA was performed using a leave-one-out cross-validation. The confusion matrix of the model indicates that the sum of the diagonal elements gave a hit rate of 100%. Eleven samples from each class were used to construct the model allowing green coffee beans with different sensorial quality to be discriminated. The confusion matrix of the PLS-DA model for green coffee beans shows that the sum of the diagonal elements gave a hit rate of 100% for high and low quality, while giving 93.8% for medium quality. The medium-quality group presented aromas from the other samples whose classification was not 100%. The rest of the samples were successfully predicted with an accuracy of 100%. Therefore, the model established that it could predict a correct discrimination of the green coffee beans of different quality.

### 3.4. Relation between E-Nose Data and Coffee Bean Aroma

**Figure 3:**
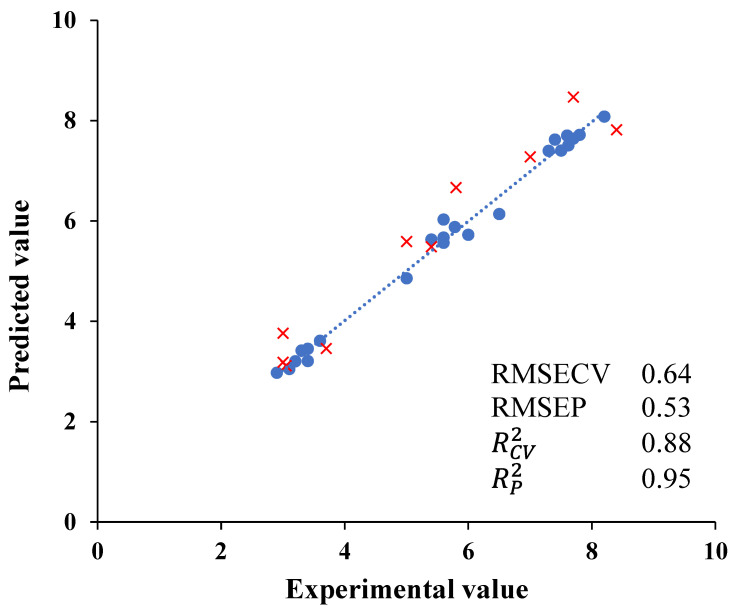
Experimental values against PLS cross-validation predictions (●) and validation set predictions (×) for fruity aroma perceived.

A multivariate analysis (PLS chemometric model) was performed between all the results obtained with the electronic nose sensors and the fruity aroma obtained by the sensory panel. These results are shown in Figure 3. This regression was used to predict the fruity aroma perceived by panelist and data from E-nose signals. $R_{CV}^{2}$ values for the models established for the fruity aroma were 0.88. Low RMSECV values of 0.64 were also estimated. The PLS calibration model was validated using samples that were not included in the calibration test. The validation parameters obtained were also acceptable. $R_{P}^{2}$ values were 0.95 for the fruity aroma perceived whilst RMSEP values were 0.53. Thus, this model led to the quantification of the fruity aroma of green coffee bean samples and its classification of different qualities. The literature points to some results related to the discrimination of roasted coffee with electronic devices. Rodríguez et al. [35] were also able to classify roasted coffee samples of different qualities using an E-nose aimed at testing the quality of the Colombian coffee. These researchers discriminated coffee with different seed defects. Gardner et al. [36], using oxide sensors, discriminated and classified three commercial coffees, as well as one coffee which had been subjected to a range of six roasting times. Brudzewski et al. [37] applied an electronic nose for the recognition of coffee with two different quality coffee brands: a mediocre product and the high-quality coffee type. Gonzalez Viejo et al. [38] estimated the intensity and aromas of roasted coffee using a low-cost and portable electronic nose and machine learning modelling.

## 4. Conclusions

Post-harvest, fresh coffee beans have different sensory characteristics that determine the quality of the final product. The positive attributes of the highest-quality coffee are linked to fruity, herbal, and sweet odors, while the negative odors are associated with roasted, smoky, earthy, and fermented odors. All these odors are represented by different VOCs. Furthermore, results obtained by the PLS models suggest that these electronic devices may be used to estimate the fruity aroma perceived by the tasting panel. The E-nose has the advantage of being an objective model with which to detect the olfactory patterns of coffee samples of different qualities. It is considered a useful discrimination tool that can be applied to green coffee beans; therefore, it can be useful for classifying coffee qualities in a production factory. This electronic device can be used in combination with a tasting panel to produce qualitative analysis, and it can be regarded as rapid, inexpensive, non-destructive, and environmentally friendly.

## Figures and Tables

**Table 1:** Results of the triangular sensory analysis test for green coffee beans (*p*-value < 0.05 for each triangle).

	High	Medium	Low
High	---	*p* < 0.05	*p* < 0.05
		*n* = 5	*n* = 5
		95%	98%
Medium	*p* < 0.05	---	*p* < 0.05
	*n* = 5		*n* = 5
	100%		95%
Low	*p* < 0.05	*p* < 0.05	---
	*n* = 5	*n* = 5	
	100%	96%	

**Table 2:** Sensory olfactory evaluation of green coffee beans (mean ± standard deviation). Different small letters indicate significant statistical differences according to the coffee qualities (Tukey’s Test, *p* < 0.05).

Quality	Positive Attributes				
	Fruity	Herbal	Sweet	Nutty	Spiced
High	7.8 ± 0.5 ^a^	4.5 ± 0.4 ^a^	3.2 ± 0.3 ^a^	2.3 ± 0.2 ^a^	2.2 ± 0.2 ^a^
Medium	5.6 ± 0.7 ^b^	3.4 ± 0.2 ^b^	2.5 ± 0.1 ^b^	1.6 ± 0.3 ^b^	1.4 ± 0.2 ^b^
Low	3.6 ± 0.4 ^c^	2.6 ± 0.3 ^c^	2.0 ± 0.1 ^b^	1.5 ± 0.2 ^b^	1.1 ± 0.2 ^b^
					
Quality	Negative Attributes				
	Roasted	Smoky	Fermented	Earthy	
High	0.6 ± 0.2 ^c^	0.5 ± 0.1 ^c^	n.d.	n.d.	
Medium	2.5 ± 0.2 ^b^	1.5 ± 0.3 ^b^	1.0 ± 0.1 ^b^	1.5 ± 0.3 ^b^	
Low	3.6 ± 0.2 ^a^	2.6 ± 0.1 ^a^	2.6 ± 0.3 ^a^	2.3 ± 0.2 ^a^	

n.d.: not detected.

**Table 3:** Content of volatile compounds (mean %, *n* = 3) obtained from green coffee beans of different qualities. R.T. = retention time. Different lowercase letters indicate significant differences (one-way ANOVA followed by Tukey’s test. *p* < 0.05) between coffee bean samples qualities.

R.T. (Min)	CAS Number	Volatile Compounds	Quantities		
			High	Medium	Low
		**Furans**			
9.0	108-29-2	Dihydro-5-methyl-2(3H)-furanone	0.70 ^a^	0.76 ^a^	0.28 ^b^
12.4	3777-69-3	2-Methyl-furan	1.04 ^b^	2.07 ^a^	2.24 ^a^
22.0	51080-20-7	Furan	0.81 ^b^	2.33 ^a^	2.27 ^a^
38.1	104-61-0	Dihydro-2-methyl-2(3H)-furanone	1.00 ^ns^	0.00	0.00
		**Pyrazines and pyridines**			
13.7	110-86-1	Pyridine	1.52 ^c^	1.94 ^b^	6.97 ^a^
15.9	75354-36-8	(E)-7-Methyl-1,6-dioxaspiro [4.5]decane	0.08 ^c^	0.26 ^b^	0.90 ^a^
24.1	24683-00-9	2,5-Dimethyl-pyridine	2.46 ^c^	3.44 ^b^	4.88 ^a^
		**Pyrroles**			
24.9	3146-84-7	3,4-Dihydro-2,2,3-trimethyl-2H-pyrrole 1-oxide	0.00 ^ns^	0.00	1.28
28.0	20189-42-8	1H-Pyrrole-2,5-dione, 3-ethyl-4-methyl-	1.31 ^a^	1.17 ^a^	0.54 ^b^
30.5		1H-Pyrrole	1.30 ^ns^	1.40	0.00
32.4	120-72-9	1H-Indole	0.00 ^ns^	0.00	0.32
		**Aldehydes**			
2.0	123-72-8	Butanal	1.49 ^ns^	0.00	0.00
2.8	96-17-3	2-Methyl-butanal	2.27 ^a^	1.27 ^c^	1.99 ^b^
5.3	66-25-1	Hexanal	2.03 ^c^	2.99 ^b^	4.29 ^a^
10.9	100-52-7	Benzaldehyde	0.00 ^ns^	0.68	0.80
16.0	2548-87-0	(E)-2-Octenal	0.00 ^ns^	0.00	0.84
18.9	124-19-6	Nonanal	7.46 ^a^	7.07 ^a^	4.17 ^b^
22.9	56114-69-3	Benzaldehyde, 2,5-bis[(trimethylsilyl)oxy]-	2.00 ^a^	1.40 ^b^	2.02 ^a^
22.6	18829-56-6	(E)-2-Nonenal	0.00 ^ns^	0.00	1.62
26.6	5910-87-2	(E,E)-2,4-Nonadienal	0.00 ^ns^	0.00	0.66
40.7	121-33-5	4-Hydroxy-3-methoxybenzaldehyde (Vanilene)	2.49 ^a^	2.78 ^a^	0.65 ^b^
		**Ketones**			
20.2	1000302-96-9	Propenone, 1-(4-nitrophenyl)-3-phenylamino-	3.17 ^ns^	0.00	0.00
27.6	2758-18-1	3-Methyl-2-cyclopenten-1-one	1.19 ^ns^	0.00	0.00
45.4	105-86-2	Geraniol	1.23 ^b^	1.71 ^a^	0.81 ^c^
71.3	502-69-2	2-Pentadecanone, 6,10,14-trimethyl-	0.73 ^c^	4.26 ^b^	5.07 ^a^
		**Esters**			
8.4	141-32-2	2-Propenoic acid, butyl ester	0.44 ^ns^	0.38	0.26
22.2	103-09-3	Acetic acid, 2-ethylhexyl ester	2.02 ^a^	1.59 ^b^	0.32 ^c^
22.2	72218-58-7	3-Methylheptyl acetate	0.84 ^ns^	0.00	0.00
26.1	109-15-9	2-Methyl-propanoic acid, octyl ester	4.33 ^ns^	0.00	0.00
54.3	1000298-25-6	1,3-Dimethylbutyl isopropylphosphonofluoridate	0.66 ^ns^	0.00	0.00
74.2	628-97-7	Hexadecanoic acid, ethyl ester	1.41 ^a^	1.43 ^a^	0.88 ^b^
80.7	544-35-4	Linoleic acid ethyl ester	0.00 ^ns^	0.53	0.59
		**Acids derivates**			
6.9	503-74-2	3-Methyl-butanoic acid	0.34 ^c^	2.32 ^b^	2.84 ^a^
6.7	4536-23-6	2-Methyl-hexanoic acid	1.51 ^a^	1.02 ^b^	0.39 ^c^
7.2	116-53-0	2-Methyl-butanoic acid	0.00 ^ns^	0.00	1.80
8.2	541-47-9	3-Methyl-2-butenoic acid	1.34 ^a^	0.69 ^b^	0.38 ^c^
		**Carboxilic acids**			
2.3	64-19-7	Acetic acid	0.12 ^c^	1.02 ^b^	1.32 ^a^
6.5	109-52-4	Pentanoic acid	0.00	0.59 ^b^	1.80 ^a^
12.1	142-62-1	Hexanoic acid	0.00 ^ns^	0.00	1.06
12.1	107-92-6	Butanoic acid	0.00 ^ns^	0.00	1.29
24.9	124-07-2	Octanoic acid	0.83 ^c^	1.99 ^b^	5.72 ^a^
31.6	112-05-0	Nonanoic acid	2.43 ^c^	3.42 ^b^	4.80 ^a^
		**Lactones**			
9.0	96-48-0	γ-Butyrolactone	0.00 ^ns^	0.00	0.39
		**Aromatics**			
12.0	108-95-2	Phenol	2.34 ^a^	1.54 ^b^	0.00
14.2	5989-27-5	(D-Limonene)	1.98 ^a^	2.01 ^a^	1.36 ^b^
17.8	90-05-1	2-Methoxy-phenol (Guaiacol)	0.66 ^ns^	0.00	0.00
34.2	7786-61-0	2-Methoxy-4-vinylphenol	9.92 ^a^	7.01 ^b^	2.27 ^c^
37.2	584-84-9	Benzene, 2,4-diisocyanato-1-methyl-	2.48 ^a^	1.79 ^b^	0.00
75.1	33777-97-8	3-Phenyl-4-azafluorene	0.00 ^ns^	0.00	0.33
80.2	15089-22-2	N-Benzyl-N-ethyl-p-isopropylbenzamide	0.00 ^ns^	0.00	1.38
		**Alcohols**			
3.8	123-51-3	3-Methyl-1-butanol,	1.51 ^b^	2.33 ^a^	2.29 ^a^
4.5	71-41-0	1-Pentanol	0.56 ^ns^	0.00	0.00
4.8	513-85-9	2,3-Butanediol	0.00 ^ns^	0.00	0.92
7.4	111-27-3	1-Hexanol	2.66 ^a^	2.38 ^b^	0.65 ^c^
11.9	3391-86-4	1-Octen-3-ol	0.96 ^b^	1.29 ^a^	1.24 ^a^
14.4	104-76-7	2-Ethyl-1-hexanol	1.81 ^a^	1.97 ^a^	1.25 ^b^
14.5	100-51-6	Benzyl alcohol	2.02 ^a^	2.06 ^a^	1.26 ^b^
19.3	60-12-8	Phenylethyl Alcohol	11.60 ^a^	11.82 ^a^	4.85 ^b^
22.0	768-95-6	1-Adamantanol	0.98 ^c^	2.14 ^a^	1.53 ^b^
23.6	143-08-8	1-Nonanol	1.07 ^ns^	0.00	0.00
26.9	122-99-6	2-Phenoxy-ethanol	1.45 ^b^	1.80 ^a^	0.67 ^c^
		**Hydrocarbons**			
18.6	78-70-6	3,7-Dimethyl-1,6-octadien-3-ol (Linalool)	0.73 ^a^	0.65 ^a^	0.36 ^b^
23.6	124-11-8	1-Nonene	0.00 ^ns^	0.00	0.90
25.7	112-40-3	Dodecane	0.00 ^ns^	0.00	0.53
41.4	629-59-4	Tetradecane	0.85 ^c^	3.55 ^a^	3.35 ^b^
		**Sulfur compounds**			
1.9	75-18-3	Dimethyl sulfide	0.00	1.98 ^b^	5.48 ^a^
		**Thiophenes**			
16.8	2557-78-0	o-Fluorothiophenol	2.88 ^ns^	2.99	0.00
25.2	1708-32-3	2,5-Dihydro-thiophene	0.33 ^c^	1.67 ^b^	2.79 ^a^
		**Others**			
8.1	100-42-5	Styrene	1.01 ^a^	0.52 ^n^	0.14 ^c^
11.6	127-91-3	Beta-Pinene	1.67 ^ns^	0.00	0.00

## Data Availability

The authors confirm that the data supporting the findings of this study are available within the article and the raw data that support the findings are available from the corresponding author, upon reasonable request.
